# A new signaling cascade linking BMP4, BMPR1A, ΔNp73 and NANOG impacts on stem-like human cell properties and patient outcome

**DOI:** 10.1038/s41419-018-1042-7

**Published:** 2018-09-27

**Authors:** Thibault Voeltzel, Mario Flores-Violante, Florence Zylbersztejn, Sylvain Lefort, Marion Billandon, Sandrine Jeanpierre, Stéphane Joly, Gaelle Fossard, Milen Milenkov, Frédéric Mazurier, Ali Nehme, Amine Belhabri, Etienne Paubelle, Xavier Thomas, Mauricette Michallet, Fawzia Louache, Franck-Emmanuel Nicolini, Claude Caron de Fromentel, Véronique Maguer-Satta

**Affiliations:** 1 CNRS UMR5286, Centre de Recherche en Cancérologie de Lyon, 69000 Lyon, France; 2 Inserm U1052, Centre de Recherche en Cancérologie de Lyon, 69008 Lyon, France; 3 Université de Lyon, 69000 Lyon, France; 4 Department of Tumor Escape Signaling, INSERM U1052, CNRS UMR5286, 69000 Lyon, France; 5 Université de Lyon 1, 69000 Lyon, France; 6 Centre Léon Bérard, 69000 Lyon, France; 7 Hospices Civils de Lyon, Hematology Department, Centre Hospitalier Lyon Sud, 69495 Pierre Bénite, France; 8 CNRS ERL 7001, 37032 Tours, France; 9 CNRS GDR 3697 MicroNiT, Tours, France; 10 Inserm, UMR1170, 94000 Villejuif, France

## Abstract

In a significant number of cases cancer therapy is followed by a resurgence of more aggressive tumors derived from immature cells. One example is acute myeloid leukemia (AML), where an accumulation of immature cells is responsible for relapse following treatment. We previously demonstrated in chronic myeloid leukemia that the bone morphogenetic proteins (BMP) pathway is involved in stem cell fate and contributes to transformation, expansion, and persistence of leukemic stem cells. Here, we have identified intrinsic and extrinsic dysregulations of the BMP pathway in AML patients at diagnosis. BMP2 and BMP4 protein concentrations are elevated within patients’ bone marrow with a BMP4-dominant availability. This overproduction likely depends on the bone marrow microenvironment, since MNCs do not overexpress BMP4 transcripts. Intrinsically, the receptor BMPR1A transcript is increased in leukemic samples with more cells presenting this receptor at the membrane. This high expression of *BMPR1A* is further increased upon BMP4 exposure, specifically in AML cells. Downstream analysis demonstrated that BMP4 controls the expression of the survival factor ΔNp73 through its binding to BMPR1A. At the functional level, this results in the direct induction of NANOG expression and an increase of stem-like features in leukemic cells, as shown by ALDH and functional assays. In addition, we identified for the first time a strong correlation between ΔNp73, BMPR1A and NANOG expression with patient outcome. These results highlight a new signaling cascade initiated by tumor environment alterations leading to stem-cell features and poor patients’ outcome.

## Introduction

The current paradigm on the initiation of leukemogenesis implies a multistep process involving different types of genetic alterations, with no obvious hierarchy and understanding of the sequential clonal selection^1^. Nevertheless, crosstalk between leukemic stem cells and the associated bone marrow (BM) stroma appears to be essential for leukemic progression and response to therapy^2,3^. More globally, understanding interactions between tumor stem cells (SCs) and their microenvironment is a challenge to develop strategies to avoid relapses after therapy. Among the main elements implicated in the crosstalk between the microenvironment and both normal and tumor SCs, we have investigated the role of bone morphogenetic proteins (BMPs), because they govern SC regulation including hematopoietic^4,5^, neural and epithelial systems^6^ by directly and indirectly affecting their niche^7–9^. Alterations of the BMP signaling pathway have been observed in numerous cancers, in some cases closely associated with cancer stem cells (CSC) properties^10^. According to the context, BMPs could participate in initial tumor suppression or favor CSC maintenance and metastasis^8^. Within the BMP family, BMP2 and BMP4 have emerged as key regulators of normal and cancer SCs^11–13^. We have previously demonstrated that alterations in the BMP pathway at intrinsic (BMP receptors and downstream partners) and extrinsic (BMP extracellular ligands) levels constitute major events in transformation, expansion and persistence of immature cells in chronic phase chronic myeloid leukemia (CML) and breast cancer, by diverting their normal functions^11,12,14,15^.

Acute myeloid leukemia (AML), the first tumor where CSCs were described^16^, is a heterogeneous disease, in which the accumulation of genetic aberrations results in the uncontrolled growth of malignant undifferentiated cells. Relapse in the first years following complete remission is prevalent and may reflect the survival of resistant immature-like tumor cells able to regenerate the entire tumor^17^. The BMP pathway has been implicated in adult AML. For example, the overexpression of the transcription factor *MIXL1*, which is sufficient to initiate AML, can be induced by BMP4. Consistent with this induction, AML cells that express *MIXL1* are sensitive to type BMP type 1 receptors (BMPR1) inhibitors^18^. In addition, in acute megakaryoblastic leukemia, the appearance of a specific fusion protein CBFA2T3-GLIS2 leads to the overexpression of BMP2 and BMP4 by leukemic cells and is associated with colony-forming capacities, a property ascribed to immature cells^19^.

Here we have identified alterations of the BMP pathway and revealed their importance in immature properties exhibited by AML cells. Initially focusing on the analysis of AML patient samples collected at diagnosis and subsequently experimentally deregulating the BMP pathway, we have identified alterations in BMP ligands, receptors and target genes. Our data highlight a new signaling cascade likely involved in the cell survival and features of immature AML cells in their microenvironment.

## Materials and methods

### Protein quantification

Bone marrow plasma obtained from allogeneic BM healthy donors and AML patients was harvested and cleared. BMPs concentration was determined using the human BMP2-ELISA or BMP4-ELISA kits (RayBiotech) following the manufacturer’s instructions.

### Primary cells, cell lines culture conditions, and treatments

Patient samples were obtained after informed consent in accordance with the Declaration of Helsinki in the hematology departments involved in this study. Mononuclear cells (MNCs) from 54 blood and BM samples were obtained from AML, excluding acute promyelocytic leukemia, patients at diagnosis. AML characteristics are presented in Table S1. Normal samples correspond to steady-state peripheral blood and BM samples from healthy donors for allogeneic BM transplantation, collected after informed consent. When necessary, primary cells were maintained in IMDM culture medium containing 10% fetal calf serum (FCS). KG1A myeloid leukemia cells were cultured in RPMI-1640 medium containing 10% FCS. BMP4 and LDN-193189 (20 nM) (Sigma-Aldrich) were added in serum-free medium as indicated^18,20^. Normal goat IgG control (AB-108-C) and anti-hBMPR1A (AF346) (R&D Systems) were used at 4 µg/mL.

### Functional assays

Colony-forming cell (CFC) and long-term culture-initiating cell (LTC-IC) assays were performed as described^20^. LTC-IC number was expressed as W5-CFC/10,000 initial cells.

### Expression vectors, transfections and luciferase assay

KG1A cells were transfected with pBabe**-**ΔNp73^21^, pMXS-NANOG or empty vector (control) (Addgene) using a Neon (Thermofisher Scientific) electroporation device according to the manufacturer’s instructions. For luciferase assays, the pNANOG-Luc reporter vector was cotransfected with empty pCDNA3 or pCDNA3-ΔNp73 in KG1A cells. Luciferase assays were performed using Dual-Glo® Luciferase Assay System (Promega) following the manufacturer’s instructions. Relative activation as compared with cells transfected with the empty vector^22^.

### RNA isolation and analysis

Quantitative RT-PCR was performed using standard protocols^20^. MNCs were isolated by a Ficoll gradient and total RNA was purified using TRI REAGENT™ (Sigma) and the mini-RNA extraction kit (Qiagen, Valencia, CA). For RT-qPCR, cDNA was produced using Superscript II (Invitrogen) and amplified using Sybr-green (Quantifast, Qiagen) and the Real-Time PCR system (Roche). TBP (TATA-binding protein) and HPRT (hypoxanthine-guanine phosphoribosyl transferase) genes were used for normalization. Arbitrary unit (AU) corresponds to the ratio of expression between samples and a single normal sample used as a reference in each PCR. Primer sequences are presented in the Table S2.

### Flow cytometry analysis

Cells were incubated with antibodies specifically recognizing CD34, CD38 (Becton Dickinson) and/or BMPR1A (R&D System), or an irrelevant isotype-matched control antibody. MNCs separated by Ficoll gradient were subjected to sorting using BMPR1A antibody and an FACSARIA III cell sorter (Becton Dickinson). ALDH (aldehyde dehydrogenase) activity was determined using an Aldefluor kit according to the manufacturer’s recommendations (StemCell Technologies).

### Western blot analysis

MNCs from AML samples were treated with BMP4 (20 ng/ml) during 24 h, then proteins were extracted. Per lane, 40 µg of proteins were loaded on SDS-PAGE and transferred onto polyvinylidene difluoride membrane (Bio-Rad). Membranes were incubated with monoclonal antibodies anti-ΔNp73 (Abcam, ab13649) or anti-βactin (Abcam, ab8226) from mouse, or anti-NANOG (Abcam, ab109250) from rabbit. Then membranes were incubated with their relevant HRP-coupled secondary antibodies (Jackson ImmunoResearch).

### Datasets

Normalized Reads Per Kilobase Million (RPKM) Illumina GA-IIX RNA-seq profiles were downloaded from the TCGA AML data portal. One hundred and sixty-one patients in the TCGA AML cohort had both RNA-seq and clinical data. RPKM values were log-transformed to the base two after adding a value of 1. Expression levels were extracted from the log-transformed data. A representative probe set with the highest average intensity was selected for each gene in each dataset. The median of expression of each gene was used for patients’ stratification (high vs. low score).

### Statistical analysis

Unless otherwise specified, statistical analysis was performed using the Mann−Whitney *U* test. Except for overall survival, the statistical analyses and graphs were performed with Graphpad prism (version 6). Significant *P* values are given in the text or symbolized by asterisks (*P < 0.05; **P < 0.01; ***P < 0.001; ****P < 0.0001). Overall survival (OS) curves from the date of sampling onwards are illustrated using the Kaplan−Meier method. Log-rank tests were conducted to evaluate the effect of markers on survival. Best cut-off values for expression of each gene of interest were determined with the Youden index computed on Cumulative/Dynamic time-dependent ROC curve using Inverse Probability of Censoring Weighting (IPCW) estimation between 12 and 36 months of follow-up. The best cut-off value for NANOG expression that discriminates the most death events during time was 13 at 24 months of follow-up (AUC = 0.77, sensitivity = 0.61/specificity = 0.85). The level of significance was set at 5%. OS analysis and graph were performed with the R program (version 3.2.3) using the “survival” and “ggplot2” packages.

## Results

### Identification of intrinsic and extrinsic alterations in the BMP pathway in AML

**Fig. 1:**
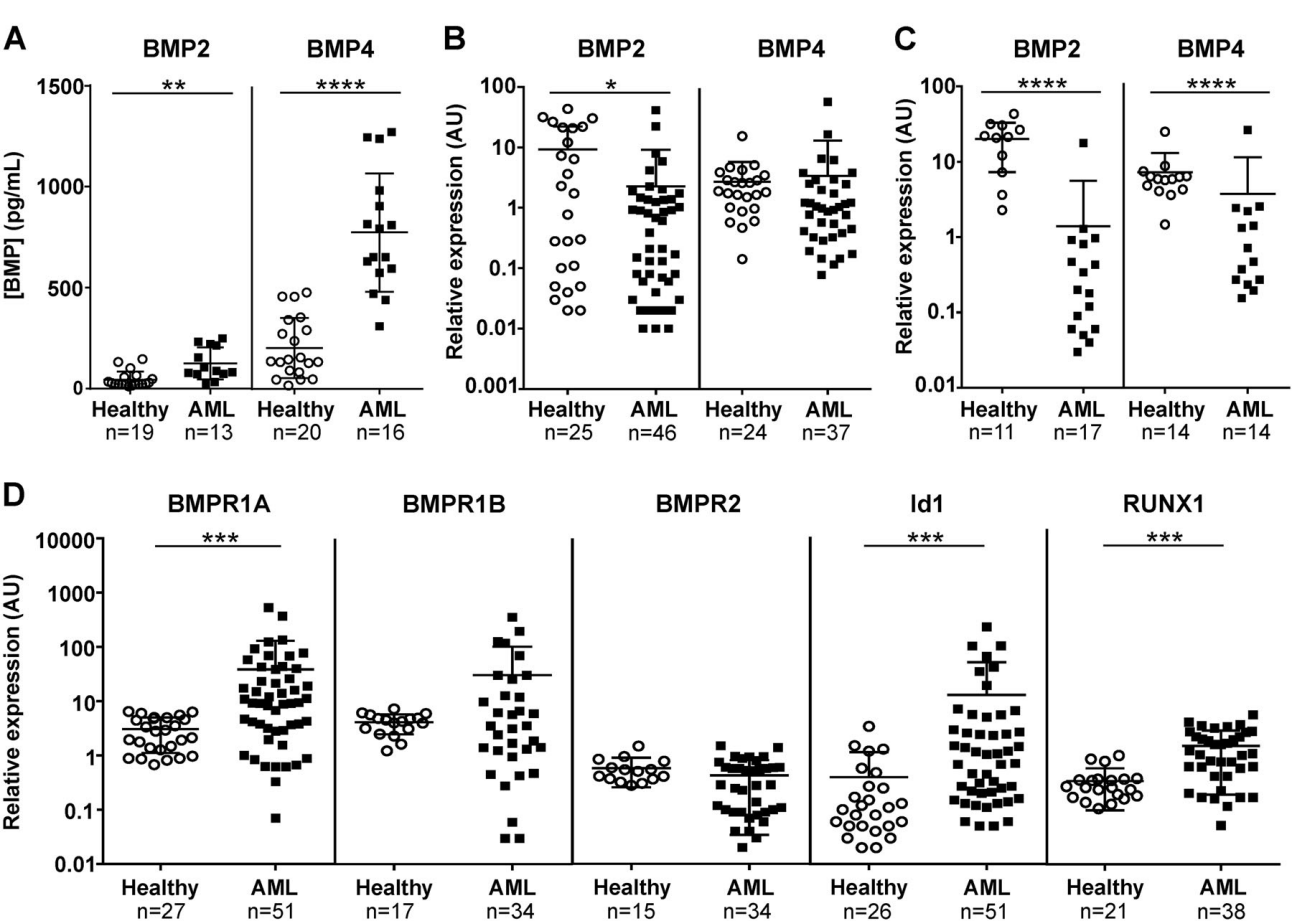
The BMP pathway is activated in bone marrow (BM) and peripheral blood of AML patients. **a** BMP2 and BMP4 concentrations in bone marrow (BM) supernatants from AML patients (*n* = 17) and healthy donors (*n* = 19) evaluated by ELISA. **b** BMP2 and BMP4 mRNA level in blood and bone marrow MNCs from healthy donors and AML patients’ samples at diagnosis. **c** BMP2 and BMP4 mRNA level in bone marrow MNCs only, from healthy donors and AML patients’ samples at diagnosis. **d** Expression of BMP receptors: BMPR1A, BMPR1B and BMPR2, and BMP target genes: Id1 and RunX1 evaluated in normal (*n* = 14–25) and AML (*n* = 34–51) grouped BM and blood samples by RT-qPCR. Arbitrary unit (AU): relative expression compared to a healthy sample used as a reference for each PCR experiment. *P* values were determined using the Mann−Whitney *U* test. *P < 0.05; **P < 0.01; ***P < 0.001; ****P < 0.0001

Elements of the BMP pathway were analyzed in AML samples (characteristics in Table S1) in both leukemic cells and their BM environment (Fig. S1a). We first measured the levels of soluble BMP2 and BMP4 in BM plasma from healthy donors and AML patients at diagnosis (Fig. S1b for experimental design). The levels of soluble BMP2 and BMP4 were significantly increased in AML patients compared to healthy donors by 2.9-fold (*P* = 0.0100) and 3.6-fold (*P* < 0.0001), respectively (Fig. 1a). Thus, in the leukemic microenvironment, cells are exposed to elevated concentrations of BMP2 and BMP4, the latter being almost sixfold more abundant than BMP2. In order to determine whether BM leukemic cells themselves produced these high BMP levels, the expression of the BMP2 and BMP4 genes was monitored in MNCs from both peripheral blood and BM of AML or healthy donors (Fig. 1b). BMP2 expression appeared reduced in leukemic cells compared to normal samples (*P* = 0.0421), while BMP4 expression was the same. Interestingly, when we analyzed BMPs transcripts in BM samples, both BMP2 and BMP4 mRNA levels were reduced in AML samples (Fig. 1c), while no significant changes were observed in circulating peripheral blood cells (Fig. S2a). These data suggest that the higher concentration of BMP2 and BMP4 in BM is not related to its autologous production by leukemic cells themselves, but is rather provided by other cells in the microenvironment.

We next analyzed gene expression profiles of intrinsic BMP pathway components in AML MNCs, with a focus on BMP membrane receptors and target genes. We observed significantly (*P* = 0.0005) higher levels of *BMPR1A* transcripts, but not of *BMPR1B* and *BMPR2*, as compared to MNCs from healthy donors (Fig. 1d). With respect to the BMP-target genes, *Id1* and *RUNX1* expression was significantly upregulated compared to the levels found in samples from healthy donors (*P* < 0.0001 and *P* = 0.0001; respectively) in leukemic cells (Fig. 1d), whereas *Id3* and *RUNX2* expression remained unchanged (Fig. S2b). The observed increases in expression were detected both in BM and peripheral blood MNCs from AML patients (Fig. S2c). These results identify intrinsic dysregulations of the BMP pathway at different levels of the signaling cascade in addition to extrinsic alterations in human primary adult AML samples at diagnosis.

### BMP4 and BMPR1A increases are associated with stemness features

**Fig. 2:**
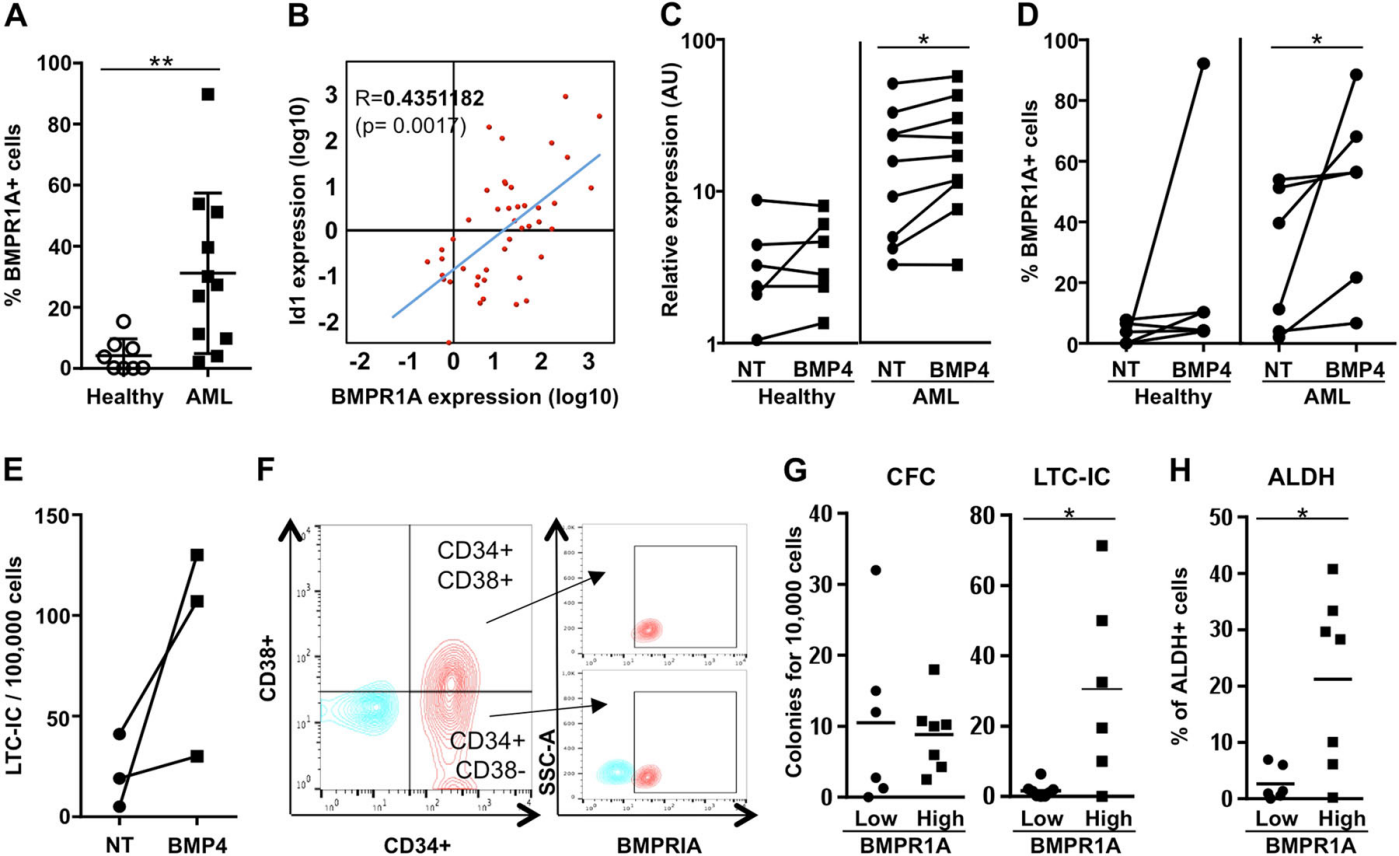
The presence of BMPR1A at the cell surface of AML samples is induced by BMP4 and BMPR1A and is associated with the presence of very immature cells. **a** Percentage of mononuclear cells (MNCs) of healthy donors (*n* = 8) and AML (*n* = 11) samples expressing BMPR1A at their membrane determined by flow cytometry. **b** Correlation between the expressions of BMPR1A and Id1 in AML samples at diagnosis (*n* = 50). **c** Relative expression of *BMPR1A* in MNCs from healthy donors (*n* = 6) and AML patients (*n* = 9), exposed or not, to BMP4 (10 ng/mL) for 24 h. **d** Effect of BMP4 (10 ng/mL) exposure on the proportion of BMPR1A-positive cells at the surface in healthy and AML MNC samples (*n* = 6 patients for each cohort). **e** Effect of BMP4 (10 ng/mL) exposure for 24 h on the proportion of very immature leukemic cells in AML mononuclear cells (MNCs), evaluated by long-term culture-initiating cells (LTC-IC) assay (*n* = 3). **f** Flow cytometric analysis of the expression of BMPR1A at the cell surface according to CD34 and CD38 status of MNCs from an AML patient. **g** Number of colony-forming cells (CFC) and LTC-IC among peripheral blood MNCs from AML patients according to their low (*n* = 8) or high (*n* = 6) *BMPR1A* mRNA level. **h** AML samples with low (*n* = 6) or high (*n* = 7) BMPR1A mRNA levels were analyzed for ALDH activity by flow cytometry. NT non-treated, NBM normal bone marrow. Statistical analysis: *P* values were determined using Spearman’s nonparametric test (**b**); Wilcoxon matched-pairs signed rank test (**c**, **d**); *P < 0.05; **P < 0.01

In order to identify BMP-responsive cells in BM, we assessed whether a correlation existed between deregulated BMP elements and the presence of BMPR1A at the cell surface. By flow cytometry analysis, we found a significantly higher number of primary MNCs overexpressing membrane-BMPR1A (BMPR1A^+^; *P* = 0.0024) in AML than in normal samples (Fig. 2a). We also observed that the expression of BMPR1A was significantly correlated with that of *Id1* in AML samples (*P* = 0.0017) (Fig. 2b) and that after sorting MNCs according to their membrane-BMPR1A content (Fig. S3a), we observed a higher *Id1* expression in BMPR1A^+^ cells (Fig. S3b). Furthermore, when we exposed healthy and AML MNCs to BMP4 for 24 h, we could observe an increase in BMPR1A expression at the transcript level in only AML MNCs (*P* = 0.0195; Fig. 2c). This was confirmed at the protein level by the increase of BMPR1A-expressing cell numbers in AML samples following BMP4 treatment (*P* = 0.0313; Fig. 2d and illustrated in Fig. S3c).

Since BMP4 is linked to the regulation of SCs^10–12,20,23^, we evaluated its involvement in immature features observed in leukemic cells. We first analyzed the effect of BMP4 exposure of AML cells on the number of long-term culture-initiating cells (LTC-IC) as a read-out of SCs properties maintenance through time (8 weeks assay). As previously reported for normal hematopoietic cells^20,24^, BMP4 treatment of AML MNCs induced a reproducible increase in the number of LTC-IC (Fig. 2e). This indicates that exposure to BMP4 favors the survival of very immature AML cells. Through cytometry analysis, we observed that BMPR1A^+^ AML cells were specifically detected in the CD34^+^CD38^−^ and CD34^+^CD38^+^ BM fractions, respectively enriched in stem and progenitor cells (Fig. 2f). We then divided at-diagnosis AML MNC samples into two groups according to their BMPR1A transcript expression. The “*BMPR1A*-low” group represented AML samples that expressed *BMPR1A* mRNA levels similar to healthy donors, while the “*BMPR1A*-high” group displayed *BMPR1A* levels exceeding the maximum value measured in healthy donors’ cells. Functional assays confirmed that AML samples expressing high levels of BMPR1A contain 15-fold more immature cells able to generate LTC-IC (*P* = 0.0426) but as many more mature progenitors identified as CFCs as BMPR1A^low^ AML samples (Fig. 2g). This was confirmed by the observation that such samples are also enriched in ALDH-positive cells (*P* = 0.0394), recognized as an SC marker in several cancer types including AML (Fig. 2h)^25,26^. Taken together, these findings indicate that leukemic cells harboring high levels of membrane BMPR1A receptors preferentially display immature features, this latter being increased after BMP4 exogenous exposure.

### BMP4 induces ΔNp73, a p53 family member associated with stemness features

**Fig. 3:**
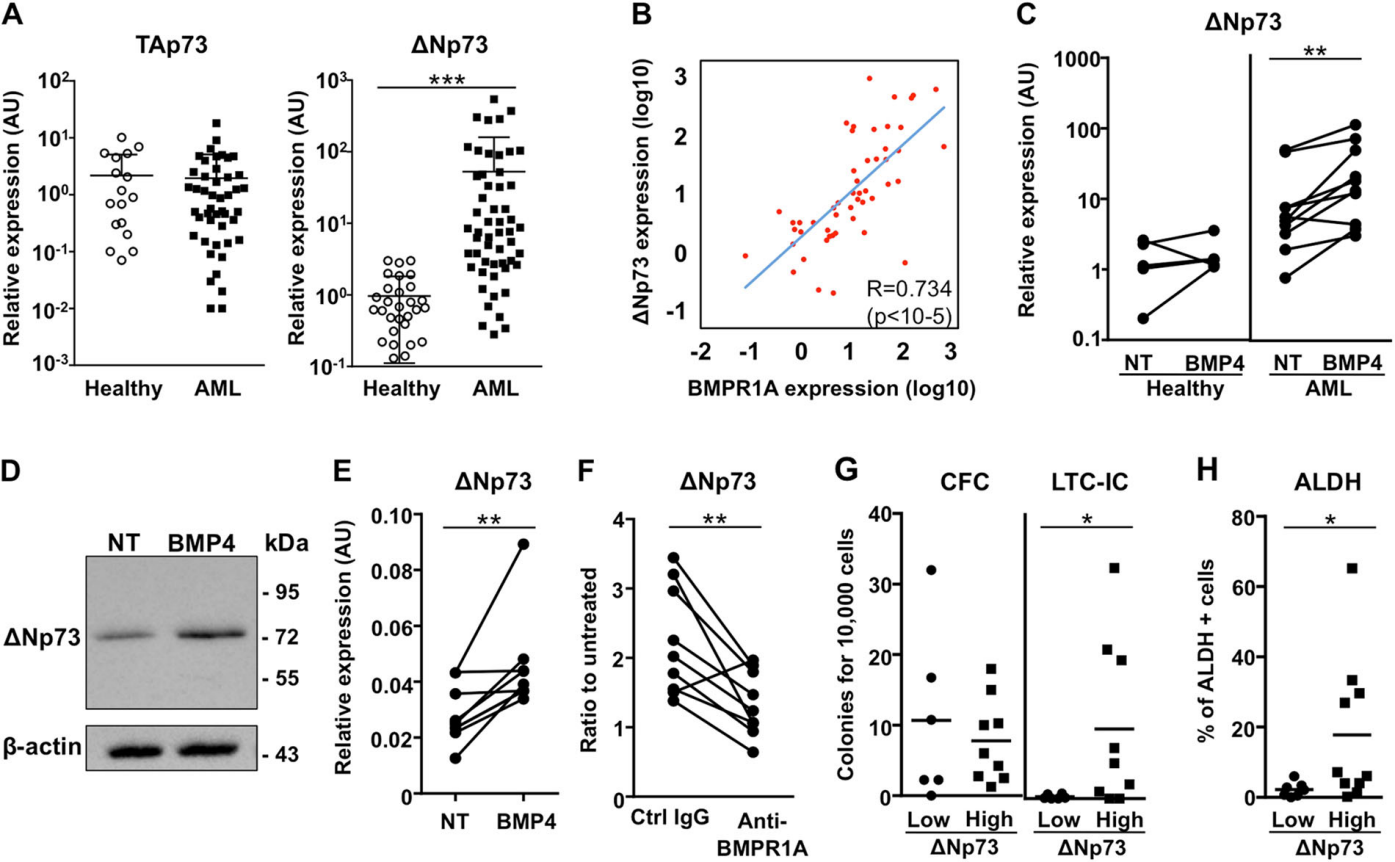
ΔNp73 overexpression in AML samples at diagnosis is correlated with BMP pathway activation and is associated with the presence of very immature cells. **a** Expression of *TAp73* and Δ*Np73* in mononuclear cell (MNC) samples from healthy donors (*n* = 19–29) and AML at diagnosis (*n* = 46–54) quantified by RT-qPCR. Arbitrary Unit (AU), relative expression compared to a healthy sample used as a reference. **b** Correlations between the expressions of Δ*Np73* and BMPR1A (*n* = 51). *P* values were determined using Spearman’s nonparametric test. **c** Relative expression of Δ*Np73* in MNCs from healthy donors (*n* = 5) and AML patients (*n* = 7), exposed or not, to BMP4 (10 ng/mL) for 24 h; NT not treated. **d** Western blot analysis of AML cells exposed or not, to BMP4 (20 ng/mL) for 24 h (*n* = 2); N not treated. **e** Relative expression of Δ*Np73* in KG1A cells exposed to BMP4 (10 ng/mL) for 24 h with (*n* = 8). **f** Expression of Δ*Np73* in KG1A cells exposed to BMP4, in presence of either an antibody specific for BMPR1A or a control antibody, measured by RT-qPCR. Ratio was calculated according to untreated KG1A cells. **g** CFC and LTC-IC values in AML samples according to low (<8 AU, *n* = 6) or high (>8 AU, *n* = 9) Δ*Np73* levels. **h** Flow cytometry analysis of ALDH activity in AML samples with low (*n* = 7) or high (*n* = 9) Δ*Np73* expressions levels. Statistical analysis: Wilcoxon matched-pairs signed rank test was used for (**c**, **e**, **f**); *P < 0.05; **P < 0.01; ***P < 0.001

BMP4, BMPR1A, and Id1 are commonly associated with cell survival probably by controlling cell proliferation and differentiation, as reported in normal and leukemic contexts^4,12,20^. In order to identify new BMP4/BMPR1A downstream partners, we evaluated the expression of other genes involved in cell survival, such as the p53 family members^27^. We focused on ΔNp73, the antiapoptotic isoform produced by the *TP73* gene^27,28^, which has been found to be overexpressed in AML patients^29,30^. We confirmed this finding in our samples where a highly significant (*P* < 0.0001) overexpression of ΔNp73 (Fig. 3a, right panel) at diagnosis, but not of the proapoptotic TAp73 isoform (Fig. 3a, left panel). Interestingly, ΔNp73 expression was strongly correlated with that of *BMPR1A* (*P* < 10^−5^) (Fig. 3b). This correlation was further confirmed by cell sorting MNCs from AML BM samples with an increase in ΔNp73 expression within the BMPR1A^+^ cells subfraction being found (Fig. S4a). Furthermore, treatment of AML or normal primary MNCs with exogenous soluble BMP4 for 24 h resulted in a significant increase in Δ*Np73* transcripts only in AML samples (*P* = 0.0059; Fig. 3c). This increase was also observed at the protein level (Fig. 3d). A similar Δ*Np73* upregulation after exposure to BMP4 was observed in the KG1A cell line, a model of immature AML^31^ (*P* = 0.0078; Fig. 3e). To confirm the direct link between BMP4, BMPR1A and the increase of ΔNp73 expression, KG1A cells were treated with BMP4 in the presence of either an anti-BMPR1A blocking antibody or the chemical BMPR1 inhibitor, LDN-193189. BMP4-mediated induction of ΔNp73 was abrogated by both the BMPR1A blocking antibody (Fig. 3f) and the LDN 193189 (Fig. S4b). Quantification of CFC and LTC-IC in two groups of samples based on their ΔNp73 expression revealed a dramatic and significant increase in LTC-IC number in the ΔNp73-high group (*P* = 0.0356) (Fig. 3g, right panel), whereas CFC remained equal (Fig. 3g, left panel). Next, we determined the number of ALDH^+^ cells in Δ*Np73-*low and -high expression groups and observed a significant correlation between Δ*Np73* expression and ALDH positivity (*P* = 0.0350; Fig. 3h). These data show that deregulation of the BMP pathway contributes to the overexpression of the survival factor Δ*Np73* in AML cells through the binding of BMP4 to BMPR1A-overexpressing leukemic cells. As this upregulation is associated with immature cell features, we next investigated potential mechanism through which ΔNp73 could participate in SC regulation.

### ΔNp73 activates *NANOG* expression in BMP4-responsive cells

**Fig. 4:**
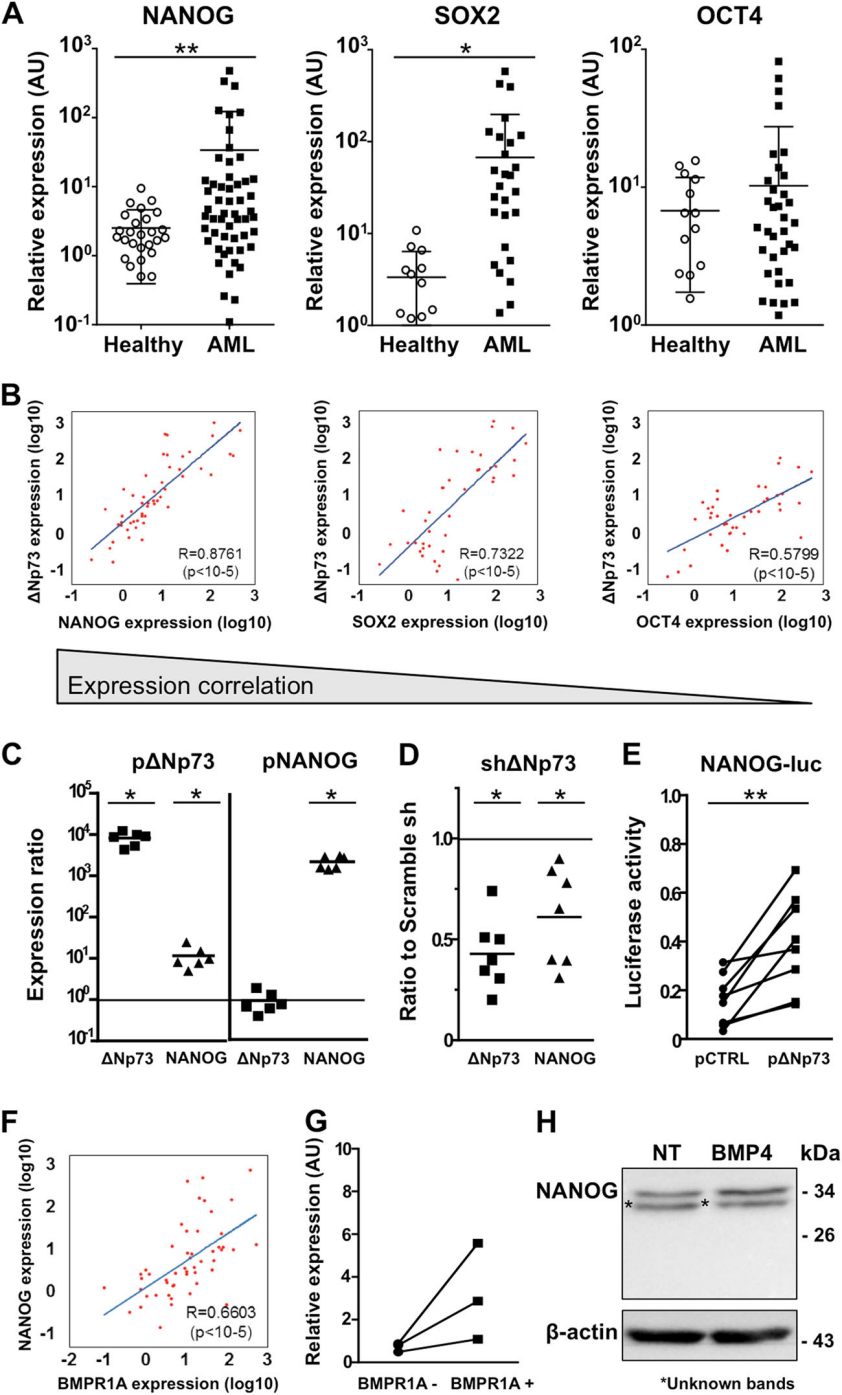
*BMPR1A*, *ΔNp73*, and *NANOG* overexpression are correlated in AML samples at diagnosis. Distribution of normal mononuclear cells (MNCs) (*n* = 14–27) and AML samples from patients (*n* = 31–54) according to **a** *NANOG, OCT4*, and *SOX2* expression. **b** Correlation between *NANOG* (*n* = 52)*, SOX2* (*n* = 41), *OCT4* (*n* = 41) and Δ*Np73* mRNA levels in AML samples. Statistical analysis: Spearman’s nonparametric test. **c** Expression of *NANOG* and Δ*Np73* in KG1A cells 48 h after transfection with pBabe-ΔNp73, pMXS-NANOG or empty vector (control). Expression ratio is the relative expression after transfection with the indicated vector as compared to transfection with the empty vector (*n* = 6). **d** Expression of *NANOG* and ΔNp73 48 h after KG1A cells transfection with an anti-ΔNp73-specific shRNA (*n* = 6). **e** Luciferase assay using the pNANOG-Luc, as reporter, cotransfected with a vector expressing ΔNp73 or an empty vector in KG1A cells (*n* = 6). **f** Correlation between *BMPR1A* and *NANOG* mRNA levels (*n* = 51). Statistical test: Spearman’s nonparametric test. **g** *NANOG mRNA levels* in AML cells sorted according to their surface expression *BMPR1A* (*n* = 3). RT-qPCR was used for mRNA quantification. Arbitrary Unit (AU), relative expression compared to a healthy sample used as a reference. **h** Western blot analysis of primary AML cells exposed or not, to BMP4 (20 ng/mL) for 24 h. Statistical analysis: *P* values were determined using Wilcoxon signed rank test for **c**, **d**; Wilcoxon matched-pairs signed rank test was used for **e**; *P < 0.05; **P < 0.01; ***P < 0.001

Since Δ*Np73* has been described as a regulator of *NANOG*^32,33^, we evaluated the potential link between Δ*Np73* and genes known to drive SC features in AML samples. For this, we analyzed the expression of the three self-renewal regulators *NANOG, SOX2*, and *OCT4*^33–35^. Although no difference was observed as compared with normal MNCs for *OCT4* (*P* = 0.5500), a significantly higher expression of *NANOG* (*P* = 0.0025) and *SOX2* (*P* = 0.0359) was measured in AML MNCs (Fig. 4a). In addition, the expression of Δ*Np73* and *NANOG* and Δ*Np73* and *SOX2* were strongly correlated (Fig. 4b). Using KG1A cells, we observed that ectopic Δ*Np73* induced *NANOG* expression (*P* = 0.0286; Fig. 4c), whereas ectopic *NANOG* did not affect Δ*Np73* expression (Fig. 4c). In addition, the reduction of Δ*Np73* expression by RNA interference consistently decreased *NANOG* expression (*P* = 0.0286; Fig. 4d). Finally, using a luciferase reporter assay, we showed that ΔNp73 is able to activate the *NANOG* promoter (Fig. 4e), as previously reported in a reprogramming iPS fibroblast model^32^.

A significant correlation between *BMPR1A* and *NANOG* expression in AML samples was also observed (*P* < 10^−5^; Fig. 4f). This link was further confirmed in BM cells isolated from three different AML samples in which we detected higher levels of *NANOG* transcripts in total MNC cells sorted only for the BMPR1A cell membrane expression (Fig. 4g). Furthermore and in agreement with the robust correlation between *BMPR1A*, Δ*Np73*, *and NANOG* expression, we observed the induction of the NANOG protein after BMP4 treatment (Fig. 4h). Lastly, treating primary AML cells by BMP4 allows correlation in the same cells of the BMP4-increased expression of *BMPR1A*, Δ*Np73*, *and NANOG* with the amplification of cells with stemness features identified by the LTC-IC assay (Supplemental Table S4). Altogether, our data indicate that BMP4 induces the expression of Δ*Np73*, which in turn, activates *NANOG* transcription.

### The combined *BMPR1A/ΔNp73/NANOG* overexpression at diagnosis identifies AML patients with a higher risk of early relapse

**Fig. 5:**
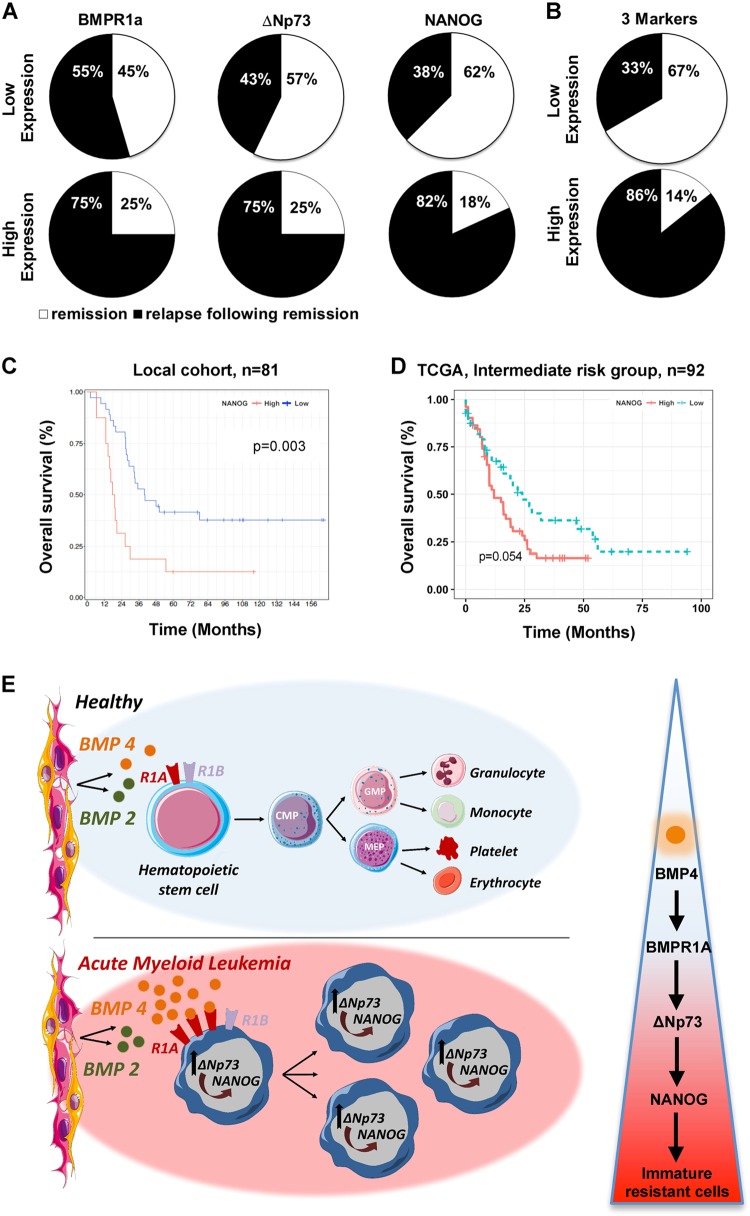
*BMPR1A*, *ΔNp73*, and *NANOG* expression at diagnosis are correlated with patient outcome. Percentage of AML patients in remission or relapse 3 years after diagnosis, according to *BMPR1A, ΔNp73* or *NANOG* expression levels separately (*n* = 19) (**a**) or in combination (*n* = 13) (**b**). **c** Overall survival (OS) curve of AML patients from the date of sampling onwards, by using the Kaplan−Meier method. The level of significance was determined by the log-rank test (*n* = 52; 23 high and 29 low NANOG level). **d** Overall survival (OS) curve of AML patients from the date of sampling onwards, by using the Kaplan−Meier method. TCGA dataset from AML patients of the intermediate group. **e** Schematic representation of the role played by BMPs on homeostasis under healthy conditions (top) and the cascade of dysregulations associated with increased concentration of BMP4 in the microenvironment of leukemic immature cells (bottom)

Finally, we analyzed the significance of *BMPR1A*, Δ*Np73*, and *NANOG* transcript levels in AML patients at diagnosis with respect to clinical outcome at 3 years post-diagnosis. We observed that levels of the three markers at diagnosis did not predict the initial complete remission status (Table S3). Conversely, high expression of either *BMPR1A*, Δ*Np73* or *NANOG* at diagnosis was associated with an increased rate of relapse within 3 years (Fig. 5a). Combining the three markers led to an increase in the clinical predictability for AML patient outcome at diagnosis, by identifying patients with a higher risk of relapse (from 33 to 86% risk of relapse; Fig. 5b). In order to evaluate the importance of NANOG, we performed a Kaplan−Meyer analysis on a larger number of patients and observed that high *NANOG* transcript levels are associated with poor survival (Fig. 5c). We performed a multivariate parameter analysis and, as expected, identified a significant correlation only between NANOG and ΔNp73 or Sox2 or Oct4 expression. No correlation with any other parameters appeared significant, as indicated in Supplemental Table S5. Lastly, a similar analysis using the independent TCGA dataset^36^, this time restricted to patients within the intermediate risk group (*n* = 92), showed again the association between high expression level of NANOG and shorter survival (*P* = 0,054). Altogether, it suggests that *NANOG* could be considered as an independent risk factor and of high interest for patient within the intermediate risk group.

These results demonstrate the importance of measuring levels of *BMPR1A*, Δ*Np73* and even more so of *NANOG*, as they could contribute to predicting at diagnosis the risk of relapse of AML patients.

## Discussion

Resistance to treatment is often associated with the persistence of cancer SCs within their microenvironment. In AML, owing to the poor clinical efficacy of current treatments, deciphering mechanisms through which the microenvironment can sustain and promote the survival of immature-like cancer cells remains an important issue for patient outcome. Among the mechanisms through which the microenvironment can affect leukemic SCs’ survival, cytokine production by stromal cells is of major importance. We focused on the BMP pathway as BMP2 and BMP4 regulate SC fate, maintenance and differentiation processes^20,24,37^ and contribute to cancer SC emergence^11,14^, maintenance and expansion^15^. Here, we found an abnormally elevated concentration of BMP4, and to a lesser extent of BMP2 in the BM plasma of AML patients. These cytokines are likely to be produced by the BM microenvironment of leukemic cells, since we did not observe any increase in BMP2 and BMP4 transcripts levels in primitive leukemic cells themselves as compared to normal hematopoietic cells. This appears to be a general mechanism as we have found similar profiles in CML at the time of diagnosis^12^ and also in luminal breast cancer^11^. Conversely, we observed an increased expression of intrinsic actors of the BMP pathway in MNCs of AML patients. In particular, we identified a highly significant increase in the expression of BMPR1A both at the mRNA and protein levels, in agreement with the important role of BMPR1 in the early steps of the AML transforming process, as previously described^18^. Interestingly, unlike the chronic phase of CML, in which we identified BMP2 and BMPR1B as driving the deregulation of the BMP pathway and SC/progenitor maintenance and expansion^12^, in AML BM cells, BMP4 and BMPR1A alterations were already detected at diagnosis. Interestingly, with CML progression towards more advanced phases and the acquisition of resistance to treatment, the BMP4 signal becomes predominant^38^. This suggests that the BMP4 signal is related to more aggressive or advanced disease as identified in cancers of other origins such as liver (HCC)^39^, thyroid (PTC)^40^ and bladder^41^. In malignant glioma, while BMP4 appears to induce differentiation of glioma cancer stem-like cells^42,43^, an epigenetic repression of the BMPR1B receptors contributes to the CSC phenotype in these tumors^10^.

We identify here that the compartment of cells expressing surface BMPR1A is enriched in very immature AML cells as demonstrated by functional and ALDH assays. In addition, an increase in LTC-IC after exposure to BMP4 confirmed the involvement of this cytokine provided by the tumor niche and of its receptor in promoting AML stem-like cells. The BM-niche was previously reported to promote survival of leukemic cells in AML by activating different cytokine-related pathways, such as SDF1/CXCR4, Wnt/β-catenin, integrins^2^. Here, the elevated concentrations of both BMP2 and BMP4 in BM of AML patients at diagnosis also support their involvement in leukemic cells survival, as described in CML^12^. These results, obtained in the context of AML, highlight the importance under pathological conditions of this pathway already found to be clearly involved in SC regulation in neural^44^, epithelial^11,14^ and hematopoietic systems^20,24^ under physiological conditions.

We investigated the interplay between BMP4/BMPR1A signaling and the ΔNp73 isoform, as this member of the p53 family has been shown to be overexpressed in AML and to regulate CSCs^23,30^. Crosstalk between the p53 family and the BMP pathway has already been reported during breast^45^ and epidermis^46^ development, as well as in tumorigenesis^47^, though no evidence for a link between BMPs and the ΔNp73 isoform has so far been reported. Here, we clearly demonstrate a functional link between BMP4 and ΔNp73. First, we identified a specific ΔNp73 upregulation already present at the time of diagnosis in MNCs of AML patients, while no such effect is seen for TAp73. Subsequently, we showed that this expression was higher within the BMPR1A^+^ subpopulation and after exposure to BMP4. Finally, we established the link between both the BMP pathway and ΔNp73 with immature cell features. Indeed, AML MNCs expressing high levels of BMPR1A and ΔNp73 were mainly ALDH positive, contained more LTC-IC and, after BMP4 treatment, exhibited an increased capacity to form colonies in an in vitro human functional SC assay.

Our results suggest that extrinsic (BMP4) and intrinsic (BMPR1A) alterations of the BMP pathway, associated with the induction of Δ*Np73* expression, contribute to the increase in the number of immature cells in AML BM. The molecular mechanisms underlying such a phenomenon were evaluated focusing on the expression of some genes associated with immaturity, in particular *NANOG*, a known ΔNp73-target gene^32,33^. We observed a significant correlation between the expression of Δ*Np73* and *NANOG* and also of *BMPR1A* and *NANOG* in AML patients. These results highlight for the first time a cascade of events in AML, initiated by the binding of BMP4 to its type 1A receptor that leads to Δ*Np73* expression, which in turn induces *NANOG* by promoter transactivation. This activation is p53-independent, since it was confirmed using the KG1A AML cell line, which does not express functional p53^31^.

The increased expression of three genes involved in self-renewal and in cell reprogramming, Δ*Np73*^32^ *NANOG* and *SOX2*^32,48^, in AML patients and the induction of Δ*Np73* and *NANOG* upon BMP4 treatment supports the hypothesis that BMP4 could promote the reprogramming of cells towards immature leukemic cells. This is consistent with a recent study that identified BMP4 and its signaling pathway as a driving element of adult cell reprogramming, including fibroblasts towards functional hematopoietic SCs^49^.

Resistance to treatment and relapse are often associated with the persistence of cancer SCs within their microenvironment. Indeed, BMP4 involvement in resistance to treatment has been demonstrated in several tumor types, including HCC^50^ and ovarian cancer^51^. BMP type 1 receptors have also been associated with resistance in CML^38^ and AML^18^, as has ΔNp73^52^. These latter, as well as *NANOG*, are also associated with resistance and progression in other cancers^53–55^.

In conclusion, our study deciphers a new signaling cascade through which alterations of BMPs secretion in the microenvironment can sustain and promote resistant immature-like leukemic cells (Fig. 5e). Activation of this signaling cascade is associated with a poor prognosis for AML patients. Thus, measuring transcript levels of the three markers *BMPR1A*, *ΔNp73*, and *NANOG* is likely to increase the ability to predict patient outcome at diagnosis. Since involvement of CSC in clinical outcome may be a common feature of relapsing cancers, it will be worthwhile to investigate the potential involvement of this novel cascade in other cancer types.

## Electronic supplementary material

Supplementary Information
