# Supplementary material for: A new signaling cascade linking BMP4, BMPR1A, ΔNp73 and NANOG impacts on stem-like human cell properties and patient outcome
Source: Cell Death Dis. 2018 Sep 27;9(10):1011. doi: 10.1038/s41419-018-1042-7 (PMC6160490; doi:10.1038/s41419-018-1042-7)
Supplement: Supplementary file 1 — Supplementary Information [file 41419_2018_1042_MOESM1_ESM.docx]

**Table S1:** Clinical data of AML patients

Prognosis groups have been defined according to international guidelines based on cytogenetic and molecular marker analyses

| **ΔNp73** | **Forward** | **AAG CGA AAA TGC CAA CAA AC** |
| --- | --- | --- |
|  | **Reverse** | **CAC CGA CGT ACA GCA TGG TA** |
| **BMP2** | **Forward** | **ACG CTC TTT CAA TGG ACG TG** |
|  | **Reverse** | **GGA AGC AGC AAC GCT AGA AG** |
| **BMP4** | **Forward** | **CTT TAC CGG CTT CAG TCT GG** |
|  | **Reverse** | **GGG ATG CTG CTG AGG TTA AA** |
| **BMPR1A** | **Forward** | **GAA AAA GTG GCG GTG AAA GT** |
|  | **Reverse** | **TAG AGC TGA GTC CAG GAA CC** |
| **BMPR1B** | **Forward** | **CTG TGG TCA CTT CTG GTT GC** |
|  | **Reverse** | **TTC CTT TCT GTG CAG CAT TC** |
| **BMPR2** | **Forward** | **TAG CAC CTG CTA TGG CCT TT** |
|  | **Reverse** | **CTG AAT TGA GGG AGG AGT GG** |
| **HPRT** | **Forward** | **CGA GCA AGA CGT TCA GTC CT** |
|  | **Reverse** | **TGA CCT TGA TTT ATT TTG CAT ACC** |
| **ID1** | **Forward** | **GGT GCG CTG TCT GTC TGA G** |
|  | **Reverse** | **TGT CGT AGA GCA GCA CGT TT** |
| **ID3** | **Forward** | **AAA TCC TAC AGC GCG TCA TC** |
|  | **Reverse** | **AAG CTC CTT TTG TCG TTG GA** |
| **NANOG** | **Forward** | **CAT CCC TGG TGG TAG GAA GA** |
|  | **Reverse** | **CCA ACA TCC TGA ACC TCA GC** |
| **OCT4** | **Forward** | **GTG AAG TGA GGG CTC CCA TA** |
|  | **Reverse** | **GAA GGA TGT GGT CCG AGT GT** |
| **RUNX1** | **Forward** | **TGG AAG AGG GAA AAG CTT CA** |
|  | **Reverse** | **CCG ATG TCT TCG AGG TTC TC** |
| **RUNX2** | **Forward** | **GTG GAC GAG GCA AGA GTT TC** |
|  | **Reverse** | **TTC CCG AGG TCC ATC TAC TG** |
| **SOX2** | **Forward** | **AAC CCC AAG ATG CAC AAC TC** |
|  | **Reverse** | **CGG GGC CGG TAT TTA TAA T** |
| **TAp73** | **Forward** | **GCA CCA CGT TTG AGC ACC TCT** |
|  | **Reverse** | **GCA GAT TGA ACT GGG CCA TGA** |
| **TBP** | **Forward** | **CAC GAA CCA CGG CAC TGA TT** |
|  | **Reverse** | **TTT TCT TGC TGC CAG TCT GGA C** |

**Table S2:** Primers used in RT-qPCR experiments

| **Initial Complete  Remission** | **BMPR1A** | | **ΔNp73** | | **NANOG** | | **3 Markers** | |
| --- | --- | --- | --- | --- | --- | --- | --- | --- |
|  | **Low** | **High** | **Low** | **High** | **Low** | **High** | **Low** | **High** |
| **Yes** | 11 (79%) | 8 (67%) | 7 (70%) | 12 (75%) | 8 (67%) | 11 (79%) | 6 (67%) | 7 (70%) |
| **No** | 3  (21%) | 4 (33%) | 3 (30%) | 4  (25%) | 4 (33%) | 3  (21%) | 3 (33%) | 3 (30%) |

**Table S3:** Number and proportion of patients that did or did not achieve complete remission after initial treatment according to expression levels at diagnosis of BMPR1A, ΔNp73, NANOG or all the 3 markers.

**Table S4:** Correlation of molecular markers induced transcriptional expression upon BMP4 treatment of primary AML cells with functional amplification of immature cells, identified by the LTC-IC *in vitro* assay. (n=4).

**Table S5:** Multivariate statistical analysis.
